# A Cross-Sectional Assessment of Nutritional Knowledge Gaps and Feasibility of Digital Intervention Among Adolescents Soccer Players in Tunisian Elite Club

**DOI:** 10.3390/nu17223598

**Published:** 2025-11-18

**Authors:** Saoussen Layouni, Sarra Ksibi, Taieb Ach, Sahbi Elmtaoua, Halil İbrahim Ceylan, Hela Ghali, Bassem Tiss, Mohamed Aziz Ajili, Sonia Jemni, Raul Ioan Muntean, Ismail Dergaa

**Affiliations:** 1 Physical Medicine and Rehabilitation Department, Sahloul Hospital, Sousse 4000, Tunisia; layouni.saoussen@famso.u-sousse.tn (S.L.); ettisbassem@gmail.com (B.T.); sonia.jemni@famso.u-sousse.tn (S.J.); 2 Faculty of Medicine of Sousse, University of Sousse, Sousse 4002, Tunisia; taieb.ach@famso.u-sousse.tn (T.A.); sahbi.elmtaoua@famso.u-sousse.tn (S.E.); hela.ghali@famso.u-sousse.tn (H.G.); 3 Laboratory of Exercise Physiology and Pathophysiology, L.R.19ES09, Sousse 4000, Tunisia; 4 Department of Family Medicine, Faculty of Medicine of Sousse, University of Sousse, Sousse 4000, Tunisia; ksibisarra@yahoo.fr; 5 Department of Endocrinology, University Hospital of Farhat Hached, Sousse 4000, Tunisia; 6 Physical Medicine and Rehabilitation Department, Ibn Eljazzar Hospital, Kairouan 3100, Tunisia; azizajili@gmail.com; 7 Faculty of Sports Sciences, Atatürk University, Erzurum 25240, Türkiye; 8 Department of Preventive and Community Medicine, Sahloul University Hospital, Sousse 4000, Tunisia; 9 Department of Physical Education and Sport, Faculty of Law and Social Sciences, University “1 Decembrie 1918” of Alba Iulia, 510009 Alba Iulia, Romania; phd.dergaa@gmail.com; 10 High Institute of Sport and Physical Education of Ksar Said, University of Manouba, Manouba 2010, Tunisia; 11 Physical Activity Research Unit, Sport and Health (UR18JS01), National Observatory of Sports, Tunis 1003, Tunisia

**Keywords:** adolescent, athletes, soccer, nutrition knowledge, sports nutrition, mobile applications, Tunisia, cross-sectional studies

## Abstract

**Background**: Adolescence represents a critical period for growth and athletic development, yet young athletes frequently demonstrate significant gaps in nutritional knowledge that can impair performance and long-term health outcomes. Limited research exists on comprehensive nutrition education interventions for adolescent soccer players in North African populations. **Objective**: To evaluate both general and sports-specific nutritional knowledge among adolescent soccer players from an elite Tunisian club and assess the feasibility of a digital nutrition intervention using mobile application technology. **Methods**: A cross-sectional survey was conducted between June and August 2024 among 50 male soccer players aged 11–18 years from Étoile du Sahel club in Sousse, Tunisia. Data were collected via a structured questionnaire comprising sections on basic nutrition knowledge, influences on food choices, sports nutrition knowledge and practices, and demographic information. A pilot digital intervention using the FatSecret app was implemented with 8 participants over 4 weeks, involving meal photo uploads and nutritionist feedback. **Results**: Participants had a mean age of 15.16 ± 1.55 years, with 92% reporting no formal nutrition education. While 90% correctly identified carbohydrates as the primary energy source, only 2% recognized that fat provides the highest energy density. Significant misconceptions existed regarding sports nutrition: 74% incorrectly believed that consuming protein 2–4 h before an event enhances performance, and only 17% knew the recommended pre-event carbohydrate intake. Food choices were primarily influenced by cravings (80%) and sensory appeal rather than health considerations (20%). The digital intervention demonstrated extremely low engagement, with minimal participation in meal photo uploads. **Conclusions**: This study reveals critical gaps in both general and sports-specific nutritional knowledge among adolescent soccer players in Tunisia, providing important descriptive information about knowledge distribution in this population. While knowledge deficits are substantial, it is important to acknowledge that this cross-sectional assessment documents only knowledge patterns, without measures of actual dietary intake or athletic performance. The persistent misconceptions and the low feasibility of the digital intervention provide important lessons regarding technology-based approaches to nutrition education in this age group, highlighting challenges in sustained engagement that must be addressed in future intervention design.

## 1. Introduction

Adolescence represents a critical developmental period characterized by rapid physical, psychological, and emotional changes that significantly impact nutritional requirements [1]. During this phase, nutrient needs increase substantially to support the growth of bones, muscles, and organs while meeting the elevated energy demands of daily activities and physical development [2]. For adolescents participating in competitive sports, proper nutrition becomes even more essential, as it supports overall growth and development, enhances athletic performance, reduces injury risk, and accelerates recovery [3].

The importance of nutritional knowledge in shaping healthy dietary habits and behaviors among young athletes cannot be overstated. A comprehensive understanding of nutrient balance, meal timing, hydration strategies, and the optimal intake of both macronutrients and micronutrients is fundamental for achieving peak performance and supporting recovery [2]. However, inadequate nutrition or reliance on inaccurate information sources can lead to dietary deficiencies that hinder growth, diminish athletic potential, and negatively affect both short-term performance and long-term health outcomes [4].

Despite the well-established importance of proper nutrition for adolescent athletes, multiple studies have identified concerning gaps in fundamental nutritional knowledge among young sports participants. A recent systematic review by Hulland et al. found that adolescent athletes demonstrate better general than sports-specific nutrition knowledge, with scores ranging from poor (33.3%) to excellent (90.6%) across studies [5]. Many adolescent athletes rely heavily on unreliable sources, including peers, coaches without specialized nutrition training, and unverified online platforms, which may provide inaccurate or misleading guidance [6]. Recent research across African populations has reinforced the importance of addressing nutritional knowledge deficits among adolescent and elite athletes [7,8]. Studies conducted in Kenya among middle- and long-distance runners revealed that although most athletes had basic nutrition awareness, over half demonstrated misunderstandings about key aspects, such as appropriate supplement use [7]. Similarly, research in Uganda observed that professional athletes frequently used dietary supplements without proper guidance, suggesting widespread gaps in practical nutrition literacy [8]. A comprehensive review examining adolescents in low- and middle-income countries highlighted systemic limitations in nutrition knowledge and dietary behavior, with particular challenges noted in urban African settings [9]. Furthermore, recent studies specifically targeting soccer players have revealed concerning knowledge gaps, with academy players demonstrating low overall sports nutrition knowledge and significant misconceptions about carbohydrate-fueling strategies [10]. This reliance on informal, potentially unreliable sources underscores the critical need to systematically assess nutritional knowledge among adolescent athletes to identify specific knowledge gaps and develop targeted educational interventions grounded in current scientific evidence.

Given identified research gaps and the limited availability of comprehensive nutritional knowledge assessments among adolescent soccer players in North Africa, our study aimed to evaluate both general and sports-specific nutritional knowledge among young athletes from a Tunisian elite soccer club. Additionally, we sought to assess the feasibility of a digital nutrition intervention using mobile application technology to improve dietary knowledge and behaviors in this population.

We hypothesized that adolescent soccer players would demonstrate significant gaps in both general and sports-specific nutritional knowledge, particularly in understanding macronutrient energy density, sports nutrition timing, and supplement efficacy. Additionally, we hypothesized that a digital nutrition intervention would show limited engagement due to the complexity of sustained behavior change among adolescents.

## 2. Materials and Methods

### 2.1. Ethical Approval

This study received approval from the local ethics committee of the Faculty of Medicine of Sousse (Ref: CEFMS 248/2024, 19 June 2024). The research was conducted in accordance with the principles outlined in the Declaration of Helsinki for research involving human participants. Written informed consent was obtained from all participants’ parents or legal guardians before study enrollment. All participants provided informed assent and were informed of their right to withdraw from the study at any time without penalty.

### 2.2. Study Design

The cross-sectional survey was scheduled during the academic summer break (June–August), coinciding with Tunisia’s academic calendar (September–June). This timing minimized overlap with examinations, reduced participant burden, and likely enhanced response rates. The intervention phase was introduced in November, during a period of seasonal stability in training schedules. By this time, athletes had settled into their routines, facilitating consistent participation without disrupting regular team activities. Participants in the intervention were selected based on confirmed availability and willingness to participate in additional study activities. All selected individuals committed to the four-week engagement period and confirmed their ability to complete the required meal photo uploads and receive nutritionist feedback. These strategic decisions supported the feasibility of recruitment for both study phases.

### 2.3. Sample Size Calculation

Sample size calculation was performed using the formula for cross-sectional studies with a single proportion:

n = (Z_1_ − α/_2_)^2^ × p × (1 − p)/d^2^

where Z_1_ − α/_2_ = 1.96 for a 95% confidence level, p = expected proportion of adequate nutritional knowledge (0.5), and d = precision (0.15). This yielded a minimum required sample size of 43 participants. To account for potential non-response, the target sample was set at 50 participants.

### 2.4. Participants

A convenience sampling method was employed to recruit 50 male adolescent soccer players aged 11 to 18 years. Inclusion criteria comprised: (1) age between 11 and 18 years, (2) active membership in the Étoile du Sahel soccer Club, (3) current participation in competitive soccer at the national level, and (4) provision of informed assent with written parental consent. Exclusion criteria included any medical condition that could interfere with participation in physical activity or dietary requirements, and the inability to complete the questionnaire due to language barriers or cognitive limitations.

### 2.5. Experimental Procedures

Since this study is based on questionnaires, we ensured the highest standards in applying psychometric methods throughout the entire study protocol, as highlighted by Guelmami et al. [11]. Data collection was conducted using a structured, self-administered questionnaire distributed through Google Forms. The questionnaire was initially developed in English based on validated instruments from established research, including tools developed by the researchers [12,13,14,15] (Supplementary Table S1). The instrument was subsequently translated into Arabic using the back-translation method and reviewed by a bilingual expert in nutrition and health education to ensure cultural appropriateness and linguistic accuracy.

Internal consistency of the questionnaire was examined using Cronbach’s alpha (α) for each major section: basic nutrition knowledge (Section A), influences on food choices (Section B), and sports nutrition-specific knowledge and practices (Section C). The α values obtained were 0.057, 0.004, and 0.189, respectively, indicating low internal consistency across sections. These results reflect the instrument’s multidimensionality and the inclusion of items with binary or categorical response formats, which are not optimal for psychometric scaling. The exploratory nature of this assessment highlights the need for further instrument refinement and validation in culturally specific contexts. Due to sample size constraints, McDonald’s omega (ω) could not be reliably estimated.

The questionnaire comprised four distinct sections: (1) Basic nutrition knowledge (11 questions focusing on macronutrients, micronutrients, and general nutritional principles), (2) Influences on food choices and availability (11 questions examining factors affecting dietary decisions), (3) Basic sports nutrition knowledge and practices (28 questions addressing performance-related nutritional concepts), and (4) Demographic and contextual information (10 questions covering participant characteristics). Response options were structured as “yes,” “no,” or “unsure” to facilitate precise categorization of knowledge levels.

Athletes completed the questionnaire under supervision, with clarifications provided when necessary to ensure an accurate understanding of the questions. All data collection sessions were conducted in a standardized manner to minimize variability in administration procedures.

### 2.6. Digital Intervention Pilot Study

An experimental sub-study was conducted in November 2024 to pilot-test the feasibility of a digital nutrition tracking intervention using the FatSecret mobile application (version 9.38.2.2). Eight participants were prospectively selected from the initial cohort for this intervention, based on predefined eligibility criteria.

Selection Methodology and Criteria:

Upon completion of the cross-sectional survey, participants who indicated interest in further involvement in the study were invited to an informational session where the intervention protocol was explained in detail, including expected time commitments, technological requirements, and data submission procedures. Of the 50 survey respondents, eight athletes fulfilled the following selection criteria: (1) demonstrated motivation, evidenced by a written statement articulating their interest and personal goals for participating; (2) verified availability for the whole 4-week intervention period, confirmed through discussions involving the athlete, and a parent or guardian; (3) possession of a compatible smartphone with confirmed installation of both WhatsApp (version 2.24.23.11) and the FatSecret application; and (4) provision of parental consent, indicating familial support and readiness to engage with the study procedures.

Pre-specified Adherence Metrics and Feasibility Endpoints:

To objectively evaluate engagement, adherence, and feasibility, a priori endpoints were defined. The primary adherence criterion required participants to upload at least two meal photographs per week (≥8 uploads over 4 weeks). Failure to meet this threshold classified a participant as ‘non-adherent,’ while meeting or exceeding it qualified as ‘adherent’.

Secondary adherence categories included: (1) ‘full adherence’, defined as ≥3 meal uploads per week (≥12 uploads total), representing documentation of approximately half of all meals; (2) ‘adequate engagement’, defined as ≥1 meal upload per week (≥4 uploads total); and (3) ‘failed engagement’, defined as <1 meal upload per week, indicating insufficient participation.

The intervention protocol included: (1) A face-to-face educational session on healthy eating and sports nutrition delivered by a certified nutritionist, (2) Parental involvement through written informed consent and creation of a WhatsApp communication group for ongoing support and technical assistance, (3) Four-week intervention period during which participants were instructed to upload photographs of breakfast, lunch, and dinner three times per week via the FatSecret app or WhatsApp, (4) Nutritional feedback provided by the certified nutritionist for each meal photograph, reviewing food choices and providing brief educational commentary.

A follow-up questionnaire was planned for the conclusion of the 4 weeks to assess changes in food habits and self-reported nutrition knowledge; however, it could not be conducted due to insufficient participant engagement.

## 3. Statistical Analysis

A primary outcome was pre-specified as achieving “adequate overall nutritional knowledge”, defined a priori as a total score ≥60% across all scored questionnaire items. Descriptive statistics were reported as means and standard deviations (SD) or percentages with 95% confidence intervals (95% CI), as appropriate.

Differences in knowledge scores were assessed between age categories (11–14 vs. 15–18 years) and educational levels using chi-square tests or independent-samples *t*-tests, depending on the data distribution. Internal consistency of each questionnaire section was assessed using Cronbach’s α.

A multivariable logistic regression model was constructed to identify predictors of adequate knowledge (yes/no), adjusting for age, training hours, and prior nutrition education. The level of statistical significance was set at *p* < 0.05. Analyses were performed using SPSS version 25.0 for Windows.

## 4. Results

### 4.1. Participant Characteristics

A total of 50 male adolescent soccer players from Étoile du Sahel soccer Club participated in this study. The mean age of participants was 15.16 ± 1.55 years, with ages ranging from 11 to 18 years. All participants competed exclusively at the national level in soccer and resided at home with their families during the study period.

Educational levels varied among participants, with the majority (36%) enrolled in the first year of high school, followed by 16% in the second year of high school, 14% in the third year of high school, 12% in the eighth year of basic education, and smaller percentages in other educational levels (Table 1). The vast majority of participants (92%) reported having received no formal education or training in nutrition or sports nutrition.

Training characteristics showed that participants engaged in an average of 5.4 ± 1.34 h of soccer training per week, with training durations ranging from 1 to 6 h. All players reported having a yearly training hiatus as part of their structured training program.

### 4.2. Basic Nutrition Knowledge

#### 4.2.1. Macronutrient Knowledge

Assessment of basic macronutrient knowledge revealed both strengths and significant knowledge gaps among participants (Table 2). Regarding carbohydrates, 90% correctly identified that carbohydrates are found in breads and cereals, and 80% recognized carbohydrates as a primary fuel source for the body. However, only 50% identified fruits and vegetables as sources of carbohydrates, and just 48% knew that carbohydrates are stored in the body as glycogen.

A critical and striking knowledge gap was identified regarding the energy density of macronutrients. 49 out of 50 participants (98%, 95% CI: 89.5–99.6%) incorrectly believed that carbohydrates contain the most energy per gram. In stark contrast, only 1 out of 50 participants (2%; 95% CI: 0.05–10.5%) accurately recognized that fat provides the highest energy content (9 kcal/g compared to 4 kcal/g for carbohydrates and protein). This near-universal misconception represents one of the most severe knowledge deficits identified in the entire assessment and demonstrates fundamental gaps in basic nutritional understanding among this population.

Protein knowledge was generally stronger, with 94% recognizing the critical role of protein in muscle recovery, 90% acknowledging that milk and dairy products contain protein, and 96% understanding that meat contains significant amounts of protein. However, 72% incorrectly believed that vegetarians cannot obtain adequate protein without consuming meat.

Fat knowledge showed mixed results: 90% correctly understood that consuming too much fat can increase body fat levels, and 72% accurately identified that most dietary fat should be unsaturated. Knowledge of specific high-fat foods was variable (Table 2), with appropriate identification of potato chips (80%) and chocolate (70%) as high in saturated fat, but confusion regarding other food sources.

#### 4.2.2. Micronutrient and Hydration Knowledge

Understanding of vitamin and mineral functions was limited, with only 36% correctly identifying that vitamins and minerals serve as catalysts for biological reactions. Knowledge of iron deficiency symptoms was better: 90% recognized tiredness, decreased athletic performance, and pale skin, though only 18% identified breathlessness as an additional symptom.

Hydration knowledge was exceptionally strong across all measured dimensions. 49 out of 50 participants (98%, 95% CI: 89.5–99.6%) correctly understood that thirst indicates dehydration, and 49 out of 50 participants (98%, 95% CI: 89.5–99.6%) correctly identified that urine color is a good indicator of hydration status. Additionally, 46 out of 50 participants (92%, 95% CI: 81.1–97.3%) recognized that water functions as a transport system in the body. These findings indicate that hydration-related knowledge represents a particular strength in this population, with the vast majority demonstrating an accurate understanding across all measured items.

### 4.3. Influences on Food Choices and Availability

Analysis of factors influencing food choices revealed that sensory and hedonic factors dominated decision-making processes. Cravings were identified as the most important factor influencing food choice by 40 out of 50 participants (80%, 95% CI: 67.0–88.8%), followed by food appearance and taste. Health considerations ranked as the primary factor for only 10 out of 50 participants (20%, 95% CI: 11.2–33.0%). This striking disparity demonstrates the dominance of hedonic factors over nutritional considerations in food choice decision-making among this population.

Parental influence ranked fourth in importance, while coach influence was ranked sixth. Cultural aspects and peer influence ranked seventh and eighth, respectively, suggesting that immediate sensory gratification takes precedence over external guidance or cultural considerations in this population.

Regarding food preparation responsibilities, 82% of participants reported that their mothers were responsible for meal preparation and grocery shopping. Only 8% of participants occasionally prepared simple dishes, such as salads, omelets, and pasta, for themselves. When asked to rate their cooking skills on a scale of 1 to 10, 56% of participants rated themselves as 5 out of 10 (Table 3).

### 4.4. Sports Nutrition Knowledge and Practices

#### 4.4.1. General Sports Nutrition Awareness

All participants (100%) agreed that nutritional guidelines differ between adolescents and adults, demonstrating awareness of age-specific nutritional needs. Additionally, 96% believed that specific nutrition strategies could enhance their athletic performance, indicating a strong motivation for nutrition education.

When asked to rate their sports nutrition knowledge on a scale of 1 to 10, 52% of participants rated themselves at 6 out of 10, indicating moderate confidence in their understanding.

#### 4.4.2. Pre-Event and During-Event Nutrition

Significant misconceptions were identified regarding pre-event nutrition strategies (Table 3). 37 out of 50 participants (74%, 95% CI: 60.4–84.1%) correctly identified the benefits of consuming carbohydrates 2–4 h before an event. However, an equal percentage, 37 out of 50 participants (74%; 95% CI: 60.4–84.1%), incorrectly believed that consuming protein 2–4 h before an event would enhance performance. Additionally, 30 out of 50 participants (60%, 95% CI: 45.9–73.0%) mistakenly thought that taking a multivitamin before an event would improve performance. These findings reveal a critical parallel between correct and incorrect beliefs regarding pre-event nutrition, suggesting that participants hold a contradictory understanding of macronutrient timing.

Knowledge of appropriate during-event nutrition was mixed. While 60% correctly identified that consuming carbohydrates during events aids performance, only 5% recognized that sports drinks could be beneficial during events. Most participants correctly avoided inappropriate options during the event, with 96% rejecting Coca-Cola and 90% rejecting fruit juice as performance aids.

#### 4.4.3. Specific Nutritional Recommendations

Knowledge of specific nutritional recommendations for athletes was limited (Table 4). Only 6% correctly understood that a banana contains 50 g of carbohydrates, with 54% expressing uncertainty about this basic nutritional fact. All participants (100%) correctly recognized that dehydration can affect sporting performance, and 64% identified blurry vision as a symptom of dehydration.

Understanding of sports drink composition was variable (Table 4), with 58% correctly identifying that sports drinks contain carbohydrates and 50% recognizing they contain salt. However, misconceptions existed, with 20% of respondents incorrectly believing that sports drinks contain fat.

Recovery nutrition knowledge was somewhat better, with participants demonstrating a good understanding of timing principles. Most recognized the importance of immediate post-exercise nutrition, though specific quantitative recommendations remained poorly understood (Table 4).

#### 4.4.4. Sports Drink Knowledge

Understanding of sports drink functions revealed both accurate knowledge and misconceptions (Table 4). Participants correctly understood that sports drinks are not superior to water (98% correct) and do not aid in burning fat (89% correct). Most also recognized that sports drinks replace sweat (74% correct) and provide water (57% correct).

However, significant knowledge gaps existed regarding the specific functional components of sports drinks. Only 4% correctly identified that sports drinks replace sodium, and just 16% recognized their role in replacing carbohydrates. Notably, 67% of respondents incorrectly believed that sports drinks replace protein.

### 4.5. Sources of Nutrition Information and Learning Preferences

Participants obtained nutrition knowledge from various sources, with parents being the most common source (28%), followed by fitness trainers (26%) and television (12%). When asked about learning preferences, 44% expressed interest in having an athlete as a guest speaker to discuss nutritional strategies, 22% were interested in interactive website programs, and 16% would appreciate lectures on nutrition advice.

Notably, 90% of participants indicated they would definitely follow the nutritional advice they received, suggesting strong motivation for behavior change if appropriate education were provided.

### 4.6. Comprehensive Statistical Analysis of Nutritional Knowledge and Associated Factors Among Adolescent Elite Soccer Players

#### 4.6.1. Prevalence and Distribution of Adequate Nutritional Knowledge Among Adolescent Athletes

The majority of adolescent soccer players (90.0%, 95% CI: 78.6–95.7%) demonstrated adequate nutritional knowledge according to our pre-specified threshold. This finding suggests that while participants possess foundational knowledge, specific misconceptions persist that require targeted intervention (Table 5). The narrow confidence interval indicates good precision in our estimate despite the relatively small sample size.

#### 4.6.2. Nutritional Knowledge Differences Between Younger and Older Adolescents Athletes

While older athletes (15–18 years) demonstrated numerically higher knowledge scores and higher knowledge rates than younger athletes (11–14 years), this difference was not statistically significant (*p* = 0.172). However, the confidence intervals suggest clinically meaningful differences may exist, with older athletes showing substantially higher rates of adequate knowledge (94.4% vs. 78.6%) (Table 6).

#### 4.6.3. Impact of Educational Level and Training Volume on Nutrition Knowledge Attainment

Similar to age stratification, participants at higher educational levels demonstrated superior nutritional knowledge, though the difference did not reach statistical significance (*p* = 0.172). The pattern mirrors age-related differences, likely reflecting the correlation between age and educational advancement in this sample (Table 7).

#### 4.6.4. Impact of Training Hours Analysis on Nutrition Knowledge Attainment

Athletes with higher weekly training hours (5–6 h) showed better nutritional knowledge compared to those with lower training hours (1–4 h), though the difference was not statistically significant (*p* = 0.164). The wide confidence interval for the low training group (45.3–93.7%) reflects the small sample size (*n* = 9) in this stratum (Table 8).

#### 4.6.5. Determinants of Adequate Nutritional Knowledge: Multivariable Logistic Regression with Adjusted Effect Estimates

The logistic regression model reveals that age is the strongest significant predictor of adequate nutritional knowledge. Each additional year of age doubles the odds of achieving adequate knowledge (OR = 2.02, 95% CI: 1.83–2.23, *p* < 0.001). Notably, training hours per week did not significantly predict knowledge levels when adjusting for other factors (OR = 1.12, *p* = 0.240). The negative coefficient for prior nutrition education likely reflects the extremely small number of participants with formal nutrition training (*n* = 4), leading to model instability for this variable (Table 9).

### 4.7. Digital Intervention Results

The 8 selected participants had a mean age of 15.3 ± 1.5 years (range: 13–17 years) and represented both younger (*n* = 2, ages 13–14) and older (*n* = 6, ages 15–17) adolescent subgroups. Educational levels ranged from 8th year (*n* = 1) to 3rd year high school (*n* = 3), with 4 participants in 1st–2nd year high school. Training hours ranged from 4 to 6 per week (mean: 5.6 ± 0.7 h), indicating consistent elite-level training engagement. No systematic demographic differences were documented between selected intervention participants and the remaining 42 survey participants.

The pilot digital intervention using the FatSecret app demonstrated poor feasibility, as indicated by pre-specified adherence metrics. No participants achieved the primary adherence target (≥2 uploads per week; ≥8 uploads over 4 weeks).

Of the eight participants selected for the 4-week intervention:

- One participant shared a single meal photograph;
- Two participants shared breakfast photographs once;
- One participant shared breakfast and lunch photographs twice;
- No participants maintained consistent engagement throughout the intervention period.

**Summary Adherence Statistics:**

- Total uploads across all participants: 5 meal photographs over 4 weeks;
- Mean uploads per participant: 0.63 ± 1.19 (median: 0; range: 0–2);
- Participants achieving primary adherence target (≥8 uploads): 0/8 (0%);
- Participants achieving secondary adherence target (≥4 uploads): 0/8 (0%);
- Participants achieving adequate engagement threshold (≥4 uploads total): 0/8 (0%);
- Participants with zero engagement: 4/8 (50%);
- Participants with 1–2 total uploads: 4/8 (50%).

Due to severely insufficient engagement with the intervention protocol, the planned post-intervention assessment questionnaire (designed to evaluate changes in self-reported nutrition knowledge and dietary practices) could not be meaningfully administered, as the intervention intensity was insufficient to expect behavioral or knowledge changes.

## 5. Discussion

This comprehensive assessment of nutritional knowledge among adolescent soccer players from an elite Tunisian club tested our pre-specified hypotheses. It revealed both expected and unexpected patterns in the distribution of knowledge. Our first hypothesis —that there are significant gaps in general and sports-specific nutritional knowledge—was accepted. Participants demonstrated critical misconceptions about macronutrient energy density (98% incorrect), sports nutrition timing (74% believed in pre-event protein benefits), and supplement efficacy (60% believed in pre-event multivitamin benefits). Our second hypothesis, concerning limited engagement with digital interventions, was also accepted, with extremely poor sustained participation despite initial enthusiasm and comprehensive support systems. However, the extent of specific knowledge gaps exceeded our initial predictions, particularly the near-universal misconception about macronutrient energy density and the prevalence of inappropriate beliefs about sports drink composition, suggesting that the knowledge deficits were more severe than hypothesized.

### 5.1. Macronutrient Knowledge Patterns and Energy Density Misconceptions

#### 5.1.1. Carbohydrate Understanding in Context

Our participants demonstrated solid understanding of basic carbohydrate sources and functions, with 90% correctly identifying cereals and breads as carbohydrate sources and 80% recognizing carbohydrates as a primary fuel source. These findings align closely with recent research by Hulland et al., who found that adolescent athletes generally performed better on general nutrition knowledge compared to sports-specific knowledge [5]. However, our findings reveal more concerning gaps than previously reported, particularly regarding misconceptions about energy density.

The critical knowledge gap identified regarding energy density—98% of participants failing to recognize that fat provides the highest energy content per gram (95% CI: 89.5–99.6%)—represents one of the most striking knowledge deficits documented in our assessment. This finding establishes a clear descriptive baseline for future intervention research. However, it is essential to note that this cross-sectional study does not verify whether knowledge of energy density is associated with actual dietary practices or athletic performance [10]. The near-universal nature of this misconception in our sample provides valuable descriptive information about knowledge patterns that warrant further investigation in prospective designs incorporating objective dietary and performance measures.

#### 5.1.2. Comparison with International Soccer Populations

Recent research on young elite soccer players across different populations has revealed varying patterns of nutritional knowledge. Studies of soccer academy players have demonstrated poor sports nutrition knowledge with significant misconceptions about carbohydrate fueling strategies [10]. Similarly, research on youth soccer players has revealed inadequate carbohydrate intake patterns, with most players consuming levels below the recommended levels for optimal performance [16]. Our findings align with these international studies, suggesting that nutritional knowledge deficits among young soccer players may be a global phenomenon rather than a region-specific issue.

However, our participants demonstrated a better understanding of basic carbohydrate functions than in some international studies, suggesting that although they possess reasonable foundational knowledge, the misconception of critical energy density represents a specific educational gap that requires targeted intervention.

#### 5.1.3. Pre-Event Nutrition Misconceptions in the Global Context

The finding that 74% of participants incorrectly believed that consuming protein 2–4 h before competition would enhance performance reflects a persistent misconception documented across multiple athlete populations. This aligns with research showing similar protein-focused misconceptions among high school soccer players, with many athletes overemphasizing protein intake at the expense of appropriate carbohydrate fueling strategies [17]. Recent studies of university athletes have shown that many incorrectly prioritize protein for immediate performance benefits over its primary role in recovery and adaptation.

Our findings are particularly concerning when compared to recent research on elite soccer academies. Academy players demonstrated similar misconceptions about pre-event nutrition, with widespread confusion about the timing and composition of pre-competition meals [10]. This consistency across different populations suggests that sports nutrition education programs globally may be inadequately addressing the distinction between immediate performance nutrition and long-term adaptation nutrition.

### 5.2. Food Choice Behaviors: International Patterns and Cultural Considerations

#### 5.2.1. Sensory Appeal Dominance Across Cultures

The predominance of hedonic factors (cravings, appearance, taste) over health considerations in our participants’ food-choice decision-making aligns closely with international research on adolescent food-choice behaviors. Studies across multiple countries have consistently shown that taste and sensory appeal are primary drivers of adolescent food choices, often overriding health considerations [18,19].

Recent research examining adolescent food choices across diverse populations found that taste preferences, convenience, and sensory appeal consistently ranked higher than health considerations, regardless of cultural background [18]. Our finding that only 20% of participants prioritized health in food selection mirrors findings from other adolescent populations, suggesting universal patterns in adolescent food choice prioritization.

#### 5.2.2. Parental Influence Patterns

The finding that parental influence ranked fourth in importance for food choice, with 82% of participants relying on their mothers for meal preparation, reflects patterns documented across Mediterranean and North African populations. Research examining soccer players found similar reliance on family food preparation, with parental influence playing a moderate but significant role in dietary choices [20,21]. However, our findings show somewhat lower parental influence compared to studies of younger adolescents, suggesting that as athletes mature, peer and personal preferences may supersede family guidance.

### 5.3. Supplement Knowledge and Misconceptions: Global Patterns

#### 5.3.1. Multivitamin Misconceptions

The finding that 60% of participants believed multivitamin supplementation before events would enhance performance reflects widespread misconceptions documented across adolescent athlete populations globally. Research examining collegiate athletes has found similar beliefs in the benefits of pre-event vitamin supplementation, despite scientific evidence showing no performance benefits in healthy, well-nourished individuals [22]. This consistency across different populations and education levels suggests fundamental misunderstandings about the role of vitamins in immediate performance versus long-term health.

Recent systematic reviews have consistently demonstrated that vitamin supplementation does not provide performance benefits in adolescent athletes, and may even be harmful when taken in excessive amounts [23,24]. However, marketing claims and informal information sources continue to perpetuate these misconceptions, as documented in studies across multiple countries and athlete populations.

#### 5.3.2. Sports Drink Confusion

The widespread misconceptions about sports drink composition, particularly the belief that sports drinks replace protein (67% of participants), represent a significant knowledge gap with practical implications. Recent research examining elite soccer players found similar confusion about the functions of sports drinks, with many athletes unclear about the specific roles of carbohydrates, electrolytes, and other components [25]. This knowledge gap is particularly concerning given the increasing use of sports drinks among young athletes and the marketing messages that may contribute to confusion about their intended purposes.

#### 5.3.3. Evidence on Nutrient Implementation and Practical Adoption Barriers

The supplement knowledge deficits documented in this study must be contextualized within the broader scientific literature on nutrient implementation and the persistent gap between evidence and practice. Substantial research describes appropriate implementation protocols and dosage requirements for achieving beneficial effects from nutrient supplementation in physically active individuals [26]. For example, L-carnitine supplementation has been shown through systematic review and meta-analysis to improve body strength, sports endurance, and exercise capacity while delaying the onset of fatigue, with established dosage protocols of 2 g/day for 4 weeks demonstrating significant changes in exercise performance markers [27]. Similarly, international sports nutrition guidelines provide evidence-based recommendations for carbohydrate timing, protein optimization, and micronutrient monitoring to support athletic performance [28].

However, despite this robust evidence base, such strategies remain not widely adopted in everyday lifestyle among adolescent athletes. Research examining supplement use patterns reveals that while 60% of adolescent athletes report using protein supplements, the majority demonstrate significant knowledge gaps regarding appropriate dosage, timing, and safety considerations [29]. This implementation gap reflects multiple factors: coaches and athletic staff often lack formal nutrition certification, adolescents lack the nutritional literacy to evaluate supplement claims critically, and commercial interests promoting supplements prioritize marketing over evidence-based application. Our findings, documenting that 60% incorrectly believed multivitamins enhance pre-event performance, 68% incorrectly believed sports drinks replace protein, and only 4% correctly identified that sports drinks replace sodium, illustrate the practical consequences of this knowledge-implementation disconnect. Without access to evidence-based guidance on nutrient supplementation, adolescent athletes remain vulnerable to non-evidence-based practices despite expressing a strong interest in performance enhancement through nutrition.

### 5.4. Nutritional Knowledge Distribution and Associated Factors: Implications for Targeted Education

The comprehensive statistical analysis extends the descriptive findings by quantifying the distribution of adequate nutritional knowledge and identifying demographic factors associated with knowledge acquisition. While 90% of participants (95% CI: 78.6–95.7%) achieved the prespecified adequacy threshold (≥60% correct), this aggregate metric must be interpreted cautiously alongside critical domain-specific deficits, particularly the 98% energy density misconception and the 74% protein timing misconception.

#### 5.4.1. Age-Related Knowledge Patterns and Developmental Considerations

The multivariable logistic regression analysis identified age as the strongest predictor of adequate nutritional knowledge, with each additional year doubling the odds of achieving it (OR = 2.02, 95% CI: 1.83–2.23, *p* < 0.001). This finding aligns with international developmental patterns, in which nutrition knowledge typically increases during adolescence through both cognitive maturation and accumulated exposure to nutrition information [30,31]. However, the magnitude of this age effect substantially exceeds that reported in comparable international research, which typically documents more modest age-related increases [5].

The age-stratification analysis revealed that older athletes (15–18 years) demonstrated numerically higher adequate knowledge rates (94.4%) than younger athletes (11–14 years; 78.6%), though this difference did not reach statistical significance (*p* = 0.172). The wide confidence interval for the younger group (52.4–92.4%) reflects the small sample size (*n* = 14) and limits the ability to draw definitive conclusions. Nevertheless, these patterns suggest that nutrition education programs should employ age-appropriate content and delivery methods tailored to developmental stages, rather than assuming uniform effectiveness across the 11–18-year range. Recent intervention research has documented that adolescent athletes respond differently to nutrition education than adults do, with interactive, developmentally appropriate approaches producing superior knowledge retention [30].

#### 5.4.2. Training Volume: Absence of Expected Association

Contrary to expectations, training hours per week did not significantly predict adequate knowledge in the adjusted model (OR = 1.12, 95% CI: 0.93–1.35, *p* = 0.240). This finding challenges the assumption that elite training environments provide incidental nutrition education through coaching interactions. The absence of a training-knowledge relationship aligns with recent international research documenting that coaches themselves often harbor significant nutrition misconceptions and lack specialized nutrition credentials [32]. This suggests that nutrition education must be deliberately structured and delivered by qualified professionals rather than assumed to occur organically through sports participation.

The stratified analysis comparing low (1–4 h/week) and high (5–6 h/week) training groups showed no significant difference in adequate knowledge rates (77.8% vs. 92.7%, *p* = 0.164). However, the small sample in the low-training stratum (*n* = 9) limits interpretation. These findings emphasize that clubs and sports organizations should implement formal, evidence-based nutrition curricula rather than relying on incidental learning through training exposure.

#### 5.4.3. Prior Nutrition Education and Systemic Gaps

The near-universal absence of formal nutrition education in this sample (92% reporting none) represents a critical systemic gap. The multivariable regression produced an unstable coefficient for prior education (OR = 0.00, *p* < 0.001) due to extreme sparsity (only 4/50 with prior education), preventing reliable inference about the relationship between education and knowledge. However, the descriptive finding itself is noteworthy: 92% of elite youth athletes in this sample have received no formal nutrition education, confirming systematic neglect of nutrition curricula within Tunisian elite youth sports settings.

International intervention research has documented substantial knowledge gains (15–30 percentage points) following structured nutrition education programs [30,33]. The extremely low baseline educational exposure in this sample suggests that even modest, systematically delivered educational interventions may produce meaningful knowledge gains. However, the cross-sectional design of this study prevents determining whether these knowledge improvements correspond to changes in dietary behavior or performance outcomes.

### 5.5. Sports Nutrition Knowledge and Digital Intervention Failures

The digital intervention using the FatSecret app demonstrated systematic failure in sustained engagement, with 50% of participants providing zero meal photographs over the 4 weeks, and no participants meeting pre-specified adherence targets (≥2 uploads per week). This represents a significant deviation from expectations for intervention feasibility and provides critical empirical data on the challenges of digital nutrition interventions in this population.

Feasibility analysis of the digital intervention revealed systemic barriers to sustained engagement among adolescent participants. These findings contribute to a growing body of evidence indicating that low adherence in adolescent-targeted digital nutrition interventions is less attributable to technological limitations and more reflective of intrinsic challenges in maintaining behavioural engagement with health-related technologies.

Three key observations support this interpretation. First, pre-intervention selection procedures, including assessments of intrinsic motivation, availability, and confirmed technological access, did not translate into continued participation, as all eight participants exhibited minimal or no adherence. This outcome suggests that neither initial motivation nor technical readiness is a reliable predictor of sustained engagement. Second, engagement was front-loaded: four meal uploads were submitted in the first week, followed by a precipitous drop-off in participation in subsequent weeks. This pattern indicates that initial curiosity or novelty may have quickly given way to disengagement, potentially due to insufficient perceived value or excessive task burden. Third, no participant responded to the nutritionist’s detailed, individualized written feedback, suggesting that the feedback mechanism was ineffective as a reinforcement tool.

These findings are consistent with international evidence on adolescent engagement with digital health interventions. Systematic reviews across health domains, including nutrition, physical activity, and mental health, report similar patterns of rapid decline in user engagement following initial uptake [34]. Importantly, these reviews emphasize that high attrition rates persist despite the inclusion of advanced features such as gamification, peer interaction, and personalized feedback [4,35]. Collectively, this evidence suggests a persistent mismatch between the design of digital health interventions, which often assume consistent user engagement, and the fluctuating motivational profiles characteristic of adolescent populations.

However, the systematic failure documented in this feasibility study provides a strong rationale for reconsidering whether digital intervention approaches are the appropriate next step for this population.

### 5.6. Limitations and Future Directions

Several limitations should be considered when interpreting these findings within the global context. The cross-sectional design prevents the establishment of causal relationships between knowledge and behavior, a limitation shared with most nutrition knowledge assessment studies [5]. The single-club recruitment limits generalizability to other athletic populations or geographic regions, though our findings align closely with international research suggesting broader applicability. The lack of test–retest assessment and incomplete omega estimation limits the robustness of instrument validation in this population.

The small sample size for the digital intervention limits conclusions about technology-based approaches, though our findings are consistent with larger studies examining digital nutrition interventions for adolescents [4,34]. Future research should employ longitudinal designs with larger, more diverse samples and include objective measures of dietary intake and nutritional status, as recommended by recent systematic reviews.

### 5.7. Integration with Global Research Trends

Our findings contribute to the growing international literature on adolescent athlete nutrition by providing data from a previously understudied North African population while demonstrating remarkable consistency with global patterns. The universality of certain misconceptions (energy density, pre-event protein beliefs, supplement efficacy) suggests that these may represent fundamental challenges in nutrition education rather than culture-specific issues.

The failure of our digital intervention aligns with emerging evidence that technology-based solutions for adolescent nutrition require more sophisticated approaches than simple app-based meal tracking [4,34,35]. This consistency across different technologies, populations, and geographic regions suggests the need for innovative intervention designs that address the complex interplay between knowledge, motivation, and sustained behavior change in adolescent populations.

### 5.8. Practical Implications and Educational Strategies

#### 5.8.1. Targeted Education Program Development

Our findings, when considered alongside international research, support the need for comprehensive, multi-component nutrition education programs designed explicitly for adolescent athletes. Recent intervention studies have shown that successful programs must address both knowledge gaps and behavioral change strategies [30]. Research has demonstrated that effective interventions combine the delivery of factual knowledge with the development of practical skills and environmental modifications.

Key educational priorities identified through our research and supported by international literature include: (1) Energy density education to correct fundamental macronutrient misconceptions, (2) Sports-specific nutrition timing and composition strategies, (3) Evidence-based information about supplement use and efficacy, (4) Practical strategies for making healthy food choices more appealing within the context of adolescent taste preferences [18,19].

#### 5.8.2. Multi-Stakeholder Involvement Strategies

The moderate influence of parents (fourth ranking) and coaches (sixth ranking) in food choice decisions suggests opportunities for multi-stakeholder educational approaches. Recent research has demonstrated that coach education programs can serve as effective multipliers for nutrition education, particularly when coaches receive evidence-based training and ongoing support [32]. Similarly, family-centered approaches have shown promise, with studies demonstrating that parental involvement significantly enhances the effectiveness of interventions.

The participants’ preference for athlete guest speakers (44%) aligns with social learning theory and peer education models, which have demonstrated effectiveness across multiple populations. Research examining elite athletes found that peer education and athlete role models were among the most effective strategies for improving nutrition knowledge and behaviors [36].

## 6. Conclusions

This study demonstrates significant deficiencies in both general and sports-specific nutritional knowledge among adolescent soccer players in Tunisia, particularly regarding macronutrient energy density, pre-exercise protein use, and supplement practices. These findings are consistent with international literature, underscoring the widespread nature of nutritional misconceptions among young athletes. The limited engagement observed in the pilot digital nutrition intervention further highlights the challenges of implementing technology-based educational strategies in this population. Despite careful participant selection and access to digital tools, sustained adherence remained minimal, suggesting that motivation and access to technology alone are insufficient to ensure effective behavioral engagement. These results emphasize the need for comprehensive, context-specific nutrition education approaches that extend beyond the transfer of factual knowledge. Interventions should incorporate behavioral strategies, involve key stakeholders, such as parents and coaches, and be tailored to adolescents’ sensory and motivational preferences. Future research should move toward prospective designs that rigorously assess the relationships among nutritional knowledge, dietary behaviors, and performance outcomes, using objective measures and more extended follow-up periods. Studies should also explore alternative mechanisms for delivering interventions, informed by qualitative insights into young athletes’ engagement patterns. Given the challenges observed with digital platforms, careful consideration should precede the implementation of technology-based solutions in this context. Instead, educational strategies should be culturally grounded and evaluated for both feasibility and effectiveness before broader application. This study provides foundational evidence to guide the development of tailored nutrition education interventions for adolescent athletes in North Africa and comparable settings. It underscores the need for multidisciplinary strategies that account for the complex determinants of dietary behavior in youth sports.

## Figures and Tables

**Table 1:** Demographics and Educational Level.

Level of Study	6th Year	7th Year	8th Year	9th Year	1st year of High School	2nd Year of High School	3rd Year High School	Baccalaureate
Headcount (%)	2%	4%	12%	10%	36%	16%	14%	6%

**Table 2:** Basic Nutrition Knowledge.

Category	Item/Statement	*n* Correct/Total	% Correct	95% CI Lower (%)	95% CI Upper (%)
Saturated Fat Knowledge					
	Almonds are high in saturated fat.	30/50	60.0%	45.9%	73.0%
	Chocolate is high in saturated fat.	35/50	70.0%	56.0%	81.7%
	Potato chips are high in saturated fat.	40/50	80.0%	67.0%	88.8%
Protein Sources					
	Baked beans contain protein	46/50	92.0%	81.3%	97.0%
	Broccoli contains protein	18/50	36.0%	23.2%	50.8%
	Chicken breast contains protein.	50/50	100.0%	92.9%	100.0%
General Nutrition Knowledge					
	Carbohydrates found in breads/cereals	45/50	90.0%	78.6%	95.7%
	Carbohydrates are stored as glycogen.	24/50	48.0%	34.4%	62.0%
	Protein is important for muscle recovery.	47/50	94.0%	83.8%	97.9%
	Vegetarians cannot get enough protein.	14/50	28.0%	17.5%	41.7%
	Thirst indicates already dehydrated.	49/50	98.0%	89.5%	99.6%

**Table 3:** Lifestyle and Sports Nutrition Practices.

Aspect	*n*/Total	%	95% CI Lower (%)	95% CI Upper (%)
Daily Eating Patterns				
Eat breakfast daily	42/50	84.0%	70.5%	91.8%
Skip meals	8/50	16.0%	8.2%	29.5%
Fast food consumption (1–2x/month)	28/50	56.0%	41.5%	69.4%
Pre-event nutrition knowledge				
Carbohydrates 2–4 h before event (correct)	37/50	74.0%	60.4%	84.1%
Protein 2–4 h before the event improves performance (misconception)	37/50	74.0%	60.4%	84.1%
Multivitamins before an event improve performance (misconception)	30/50	60.0%	45.9%	73.0%
Water immediately before the event (correct)	39/50	78.0%	64.6%	87.4%
During-Event Nutrition Knowledge				
Carbohydrates during the event aid performance	30/50	60.0%	45.9%	73.0%
Sports drinks are beneficial during an event	3/50	6.0%	1.3%	16.2%
Coca-Cola was inappropriate during the event (correct rejection)	48/50	96.0%	86.3%	98.7%
Cooking and Food Preparation				
Rate cooking skills 5/10	28/50	56.0%	41.5%	69.4%
The mother is responsible for meal preparation	41/50	82.0%	68.6%	90.6%
Occasionally prepare simple dishes	4/50	8.0%	2.2%	19.4%

**Table 4:** Specific Sports Nutrition Knowledge and Recommendations.

Statement/Question	*n* Correct/Total	% Correct	95% CI Lower (%)	95% CI Upper (%)
Specific Nutritional Facts				
50 g carbohydrate in a banana	3/50	6.0%	1.3%	16.2%
Dehydration affects sporting performance	50/50	100.0%	92.9%	100.0%
Blurry vision is a dehydration symptom	32/50	64.0%	50.0%	76.6%
Sports Drink Composition Knowledge				
Sports drinks contain carbohydrates	29/50	58.0%	43.9%	71.2%
Sports drinks contain salt	25/50	50.0%	36.4%	63.6%
Sports drinks replace protein (MISCONCEPTION)	34/50	68.0%	54.0%	79.9%
Sports drinks are better than water (MISCONCEPTION)	1/50	2.0%	0.05%	10.5%
Sports drinks help burn fat (MISCONCEPTION)	5/50	10.0%	3.5%	21.8%
Sports drinks replace sweat	37/50	74.0%	60.4%	84.1%
Sports drinks replace sodium (CORRECT)	2/50	4.0%	0.5%	13.6%
Performance Enhancement Claims				
Caffeine improves concentration	38/50	76.0%	62.6%	85.9%
Salami & eggs before the event is ideal (MISCONCEPTION)	16/50	32.0%	20.1%	46.1%
Specific Quantitative Knowledge				
Recommended carbs before competition (CORRECT VALUE)	9/50	18.0%	9.0%	30.5%
Water per hour moderate exercise (CORRECT VALUE)	11/50	22.0%	12.0%	35.1%
Carbohydrate per hour > 1 h exercise (CORRECT VALUE)	12/50	24.0%	13.7%	37.3%
Recovery snack timing—immediately post-event	44/50	88.0%	76.5%	94.8%

**Table 5:** Primary Outcome Summary.

Metric	Value	95% CI	Notes
Primary Outcome			
Adequate knowledge (≥60% threshold)	90.0%	78.6–95.7%	45/50 participants
Knowledge Score Distribution			
Mean score (out of 12)	9.52 ± 1.66		Based on a 12-item composite score
Mean percentage	79.3 ± 13.8%		
Median percentage	83.3%		
Knowledge Categories			
Poor (<40%)	1 (2.0%)		
Fair (40–59%)	4 (8.0%)		
Good (60–79%)	16 (32.0%)		
Excellent (≥80%)	29 (58.0%)		

**Table 6:** Age Group Comparison Analysis.

Age Group	*n*	Mean Score ± SD	Mean %	*n* Adequate	Adequate %	95% CI
11–14 years	14	8.86 ± 2.25	73.8%	11	78.6%	52.4–92.4%
15–18 years	36	9.78 ± 1.31	81.5%	34	94.4%	81.9–98.5%

Statistical Test: Mann–Whitney U = 189.5, *p* = 0.172.

**Table 7:** Educational Level Comparison.

Education Level	*n*	Mean Score ± SD	Mean %	*n* Adequate	Adequate %	95% CI
Middle/College (6–9)	14	8.86 ± 2.25	73.8%	11	78.6%	52.4–92.4%
Secondary/High (1–4)	36	9.78 ± 1.31	81.5%	34	94.4%	81.9–98.5%

Statistical Test: Mann–Whitney U = 314.5, *p* = 0.172.

**Table 8:** Training Hours Comparison.

Training Hours	*n*	Mean Score ± SD	Mean %	*n* Adequate	Adequate %	95% CI
Low (1–4 h)	9	8.56 ± 2.46	71.3%	7	77.8%	45.3–93.7%
High (5–6 h)	41	9.73 ± 1.38	81.1%	38	92.7%	80.6–97.5%

Statistical Test: Mann–Whitney U = 130.0, *p* = 0.164.

**Table 9:** Multivariable Logistic Regression Results.

Predictor	Coefficient (β)	SE	Odds Ratio	95% CI	*p*-Value
Intercept	6.437	0.056	624.48	559.55–696.95	<0.001
Age (years)	0.703	0.049	2.02	1.83–2.23	<0.001
Training hours (per week)	0.113	0.096	1.12	0.93–1.35	0.240
Prior nutrition education (yes vs. no)	−15.015	0.174	0.00	0.00–0.00	<0.001

## Data Availability

The datasets generated and analyzed during the current study are not publicly available due to participant privacy and ethical restrictions; however, they are available from the corresponding author upon reasonable request.

## Supplementary Materials

The following supporting information can be downloaded at: https://www.mdpi.com/article/10.3390/nu17223598/s1. Table S1: Provenance of questionnaire items by section and original source.
