# Supplementary material for: A Cross-Sectional Assessment of Nutritional Knowledge Gaps and Feasibility of Digital Intervention Among Adolescents Soccer Players in Tunisian Elite Club
Source: Nutrients. 2025 Nov 18;17(22):3598. doi: 10.3390/nu17223598 (PMC12654968; doi:10.3390/nu17223598)
Supplement: Supplementary file 1 [file nutrients-17-03598-s001.zip › nutrients-3958697-supplementary.pdf]

Supplementary Table S1

Provenance of questionnaire items by section and original source

Section	Topic	Item (Question) identifiers*	Adapted from	Reference (Vancouver style)
A – Basic Nutrition Knowledge	Macronutrients, hydration, dietary sources	A1, A2, A3, …	General Nutrition Knowledge Questionnaire (GNKQ)	Parmenter K, Wardle J. Development of a general nutrition knowledge questionnaire for adults. Eur J Clin Nutr. avr. 1999;53(4):298-308.
B – Influences on Food Choice & Eating Behaviour	Meal patterns, fast food frequency, and family food environment	B1, B2, B3, …	Adolescent Food Habits & Nutrition Knowledge Questionnaire	Turconi G, Celsa M, Rezzani C, Biino G, Sartirana MA, Roggi C. Reliability of a dietary questionnaire on food habits, eating behaviour and nutritional knowledge of adolescents. Eur J Clin Nutr. juin 2003;57(6):753-63.
C – Sports Nutrition Knowledge & Practice	Fueling around training, pre-/post-exercise intake, hydration for sport	C1, C2, C3, …	Sports Nutrition Knowledge Questionnaires	Tam R, Gifford JA, Beck KL. Recent Developments in the Assessment of Nutrition Knowledge in Athletes. Curr Nutr Rep. 1 juin 2022;11(2):241-52.
C – Sports Nutrition Knowledge & Practice	Supplements, competition nutrition, behavior	C11, C12, C13, …	Elite Athlete Dietary Intake Survey	Burkhart SJ, Pelly FE. Dietary Intake of Athletes Seeking Nutrition Advice at a Major International Competition. Nutrients. 14 Oct 2016;8(10):638. competition. Nutrients. 2019;11(10):2399.
